# An automated A-value measurement tool for accurate cochlear duct length estimation

**DOI:** 10.1186/s40463-018-0253-3

**Published:** 2018-01-22

**Authors:** John E. Iyaniwura, Mai Elfarnawany, Hanif M. Ladak, Sumit K. Agrawal

**Affiliations:** 1 Biomedical Engineering Graduate Program, Western University, 1151 Richmond Street, London, ON N6A 3K7 Canada; 2 Department of Otolaryngology-Head and Neck Surgery, Western University, London, ON Canada; 3 Department of Medical Biophysics, Western University, London, ON Canada; 4 Department of Electrical and Computer Engineering, Western University, London, ON Canada; 5 London Health Science Centre, Room B1-333, University Hospital, 339 Windermere Rd., London, ON Canada

**Keywords:** Cochlear duct length, A-value, Computer tomography, Atlas-based registration, Cochlear implants

## Abstract

**Background:**

There has been renewed interest in the cochlear duct length (CDL) for preoperative cochlear implant electrode selection and postoperative generation of patient-specific frequency maps. The CDL can be estimated by measuring the A-value, which is defined as the length between the round window and the furthest point on the basal turn. Unfortunately, there is significant intra- and inter-observer variability when these measurements are made clinically. The objective of this study was to develop an automated A-value measurement algorithm to improve accuracy and eliminate observer variability.

**Method:**

Clinical and micro-CT images of 20 cadaveric cochleae specimens were acquired. The micro-CT of one sample was chosen as the atlas, and A-value fiducials were placed onto that image. Image registration (rigid affine and non-rigid B-spline) was applied between the atlas and the 19 remaining clinical CT images. The registration transform was applied to the A-value fiducials, and the A-value was then automatically calculated for each specimen. High resolution micro-CT images of the same 19 specimens were used to measure the gold standard A-values for comparison against the manual and automated methods.

**Results:**

The registration algorithm had excellent qualitative overlap between the atlas and target images. The automated method eliminated the observer variability and the systematic underestimation by experts. Manual measurement of the A-value on clinical CT had a mean error of 9.5 ± 4.3% compared to micro-CT, and this improved to an error of 2.7 ± 2.1% using the automated algorithm. Both the automated and manual methods correlated significantly with the gold standard micro-CT A-values (*r* = 0.70, *p* < 0.01 and *r* = 0.69, *p* < 0.01, respectively).

**Conclusion:**

An automated A-value measurement tool using atlas-based registration methods was successfully developed and validated. The automated method eliminated the observer variability and improved accuracy as compared to manual measurements by experts. This open-source tool has the potential to benefit cochlear implant recipients in the future.

## Background

Cochlear implants (CI) are now commonly used worldwide to restore hearing in patients with severe to profound sensorineural hearing loss (SNHL) [1–3]. The literature has described significant variation in the human cochlear duct length (CDL) [4–8],which may have an impact on CI electrode selection for patients [9, 10]. An electrode cannot be too long (which would result in incomplete insertion), or too short (poorer cochlear coverage). Knowledge of the CDL a priori would help with pre-operative electrode selection.

Several studies have examined the benefits of deep insertions and complete cochlear coverage. Roy et al. reported a benefit in musical appreciation with deeper insertions [11] while Qi et al. highlighted similar benefits with tonal language discrimination [12]. Mistrik and Jolly emphasized that low frequency information delivered to the cochlear apex is particularly important for spatial hearing [13]. In addition, they commented that the matching of the electrode array length and cochlear length is the most important factor which directly affects the mapping of CI electrode array to the auditory nerve of the cochlea. Apart from lower frequency information, Hochmair et al. [10] noted that deeper insertions provide the opportunity for: 1) better mapping of tonotopic locations within the cochlea, 2) increase in the coverage of cochlear locations, and 3) the reduction of potential channel overlap due to larger contact divisions per channel.

In addition to the *pre-operative* electrode selection to maximize cochlear coverage, the CDL can be used *post-operatively* to create custom frequency maps for patients, potentially reducing place pitch mismatch, the effect of which has been studied clinically. The greenwood equation is used as a guideline for post-operative frequency mapping of the CI electrode arrays and it is directly dependant on the CDL. Based on this equation, Koch et al. demonstrated a CDL length mismatch of 6 mm would translate to a frequency mismatch of 1100 Hz in the basal region and 400 Hz in the apical region [14–16]. Fu et al. carried out a clinical study that showed a shift of just 2–4 mm affected patients’ rehabilitation times [17]. With bilateral CI users, Kan et al. and Stelmach et al. highlighted that a place mismatch can occur between the left and right electrode arrays and that this mismatch can simply be as a result of insertion depth differences [18, 19]. Studies have also shown that such place pitch mismatches can lead to poor speech recognition in noisy environments [20], a shift in the perceive location of sound sources [18], and poor interaural time differences (ITD) [21, 22]. In addition, with regards to cochlear implant users with single sided deafness (SSD), Rader et al. concluded that place dependant stimulation can be expected to improve pitch perception [23]. Other studies have shown conceivable shortcomings in CI performances due to significant levels of place pitch mismatching [15, 24–26]. In order to reduce pitch place mismatch, CDL estimates would be needed to create custom frequency maps.

The complete effects of deep insertions, frequency place pitch mismatch, and the potential benefits of customizable electrode lengths and individualized frequency map fitting, is still an ongoing active area of research, and preliminary results hold significant clinical relevance [4, 27–30].

Currently the CDL can be estimated using the A-value; a measurement defined as the length of the straight line between the middle of the round window, passing through the modiolar axis, and reaching the furthest point on the basal turn [31]. This measurement was proposed by Escudé et al., who utilized the correlation between the A-value and the CDL [31]. Alexiades et al. [32] proposed an equation to determine CDLs using the A-value, and these equations were further modified using high resolution imaging by Koch et al. [14]. However, despite the simplicity of this method and its relevance, there is significant inter-observer and intra-observer variability associated with the A-value measurement on clinical CT scans [33, 34]. The development of an automated tool to measure the A-value could alleviate this user variability.

The primary objective of this study is to develop an automated algorithm using atlas-based registration techniques on an open-source platform. The secondary objective is to compare the accuracy of the automated tool against manual measurements by experts. A set of micro-CT (*μ*CT) images of the same sample set was used as the gold standard for measurement.

## Methods

### Image acquisition

Twenty fixed cadaveric temporal bone specimens were obtained for the study. Ethics approval was acquired through the Department of Anatomy at the Schulich School of Medicine and Dentistry at Western University, Ontario Canada.

### Clinical CT images

All 20 specimens were scanned at a clinical resolution of 600 μm using the Discovery CT750 HD Clinical Scanner (GE Healthcare, Chicago, IL), equipped with GE’s Gemstone CT detector. The Scanner was set to a slice thickness of 0.625 mm and an x-ray voltage of 120 kV. The acquisition time for each of the 20 specimens was approximately 20 s.

### Micro-CT images

High resolution micro-CT (*μ*CT) images were acquired for all 20 specimens. The temporal bone specimens where trimmed using a cylindrical drill bit, with a diameter of 40 mm and a height of 60 mm. Special care was taken to ensure the region of interest was preserved. The trimmed specimens could then be imaged with the eXplore Locus *μ*CT scanner (GE Healthcare, Chicago, IL), which was set at 80 kV and 0.45 mA. Using an incremental angle of 0.4 degrees, approximately 900 views could be captured. A modified cone beam algorithm [35] was used to reconstruct a 3D image with a voxel size of 20*μ*m.

### Gold standard values

A fellowship trained neurotologist (SKA) measured the A-value on a set of 20 high resolution *μ*CT images. These images were reconstructed at an oblique angle that enabled the full basal turn of the cochlea to be visualized. The reconstructed views were subsequently displayed with an appropriate minimum-intensity projection (MinIP) as described by Escudé et al. [31]. The A-value for all 20 specimens served as the gold standard reference values [33]. As a note, intra- and inter-observer variation was insignificant on these high-resolution *μ*CT scans as the round window membrane and outer cochlear wall were clearly visible on the MinIP projections.

### Atlas generation

**Fig. 1:**
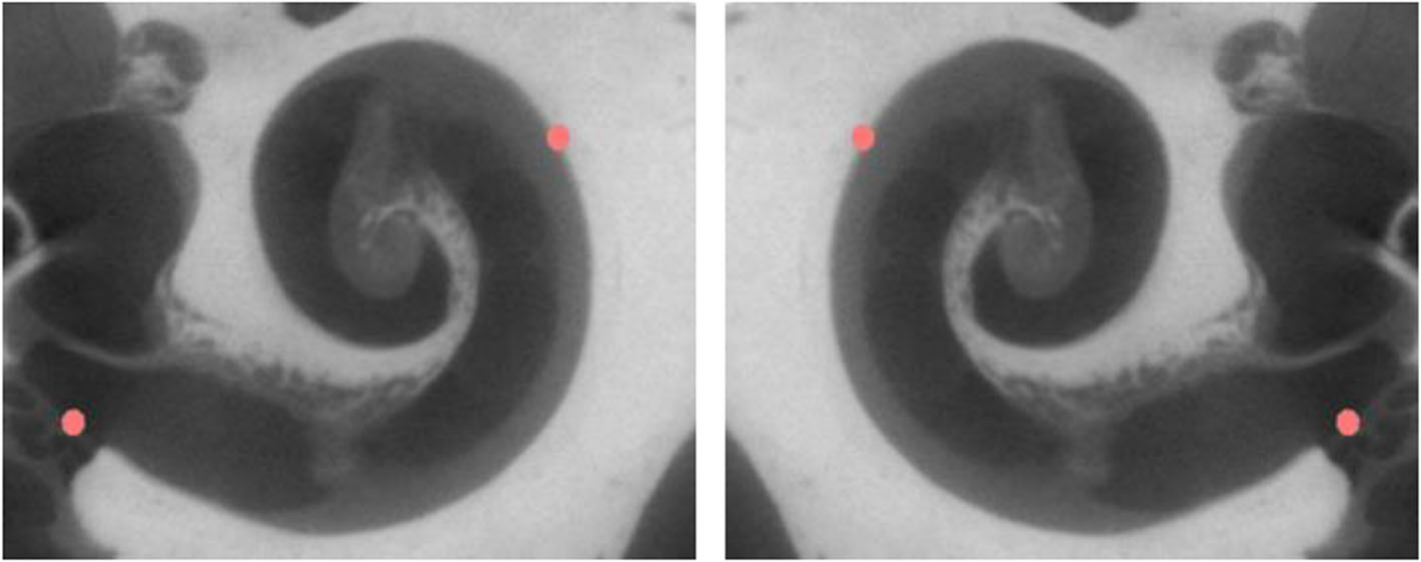
Atlas with two fiducials on the right and left cochleae

The *μ*CT image of the specimen with the median gold standard A-value was selected to be used as the single atlas for the automated algorithm. The atlas was mirrored to ensure that models were available for both right and left cochleae. To facilitate accurate registration, the atlas was cropped to only contain the region of interest in 3D Slicer [36]. Two fiducials were then placed; one on the centre of the round window and the other on furthest point on the basal turn as shown in Fig. 1.

### Registration algorithm

**Fig. 2:**
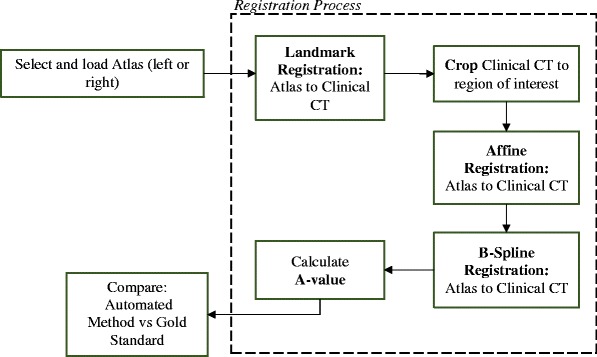
Automated A-value registration algorithm

The registration algorithm was developed on an open source software platform, 3D Slicer [36, 37]. The algorithm components are illustrated in the flowchart (Fig. 2). The atlas (source image) was loaded along with the clinical CT (target) image. Fiducials were placed on the following landmarks: the cochlear apex, modiolus, round window and oval window. Landmark registration was then used to ensure the source and target images were in the same spatial region. Finally, the target image was cropped to extract the region of interest (i.e., the cochlea and immediate surrounding structures).

### Affine registration

Affine image registration is a form of linear registration that incorporates translation, rotation, scaling, and shearing. The dimension of the image determines the degrees of freedom in the registration [38]. The CT images were 3-dimensional, which resulted in a total of 12 degrees of freedom (DOF) (translation, rotation, scaling, and shearing were performed in each of the x, y, and z axis). Affine registration is restricted in that it only captures global differences between images, therefore, it is typically used as a technique to align a set of images before non-linear registration techniques are applied [38, 39]. To capture global differences alone, only 0.1% of the clinical CT (target image) was considered by the algorithm. Normalized cross correlation (NCC) was used as the image similarity comparison metric, which defined the registration’s objective function. Table 1 outlines the complete set of parameters used.

**Table 1:** Automated method parameters for Landmark, Affine, and B-Spline registration

Registration	Initialization	Objective function	Degrees Of Freedom (DOF)	% of Sample
Landmark	Fiducial placements	Least squares	6 DOF	N/A
Affine	Geometric alignment	Normalized Cross Correlation (NCC)	12 DOF	0.1
B-Spline	Affine transform	Normalized Cross Correlation (NCC)	64 DOF	100

### B-Spline registration

A free-form deformation (FFD) model based on b-splines, was used to address local differences between the images. FFDs allows for local deformation of an image through the manipulation of a mesh of control points [39]. After the movement of the control points, a b-spline function is used to interpolate the corresponding movement in the image and the degrees of freedom for b-spline registration is determined by the number of control points [40]. After the affine registration addressed global differences, b-spline registration was then used to address local differences between the images. The parameters used for the b-spline registration in Table 1 were based on Elfarnawany et al. [41]. A 3D mesh of control points (4 x 4 x 4) allowed for a total of 64 DOF and NCC again was used as the image similarity metric used to define the objective function. The whole clinical sample (100%) was used in the registration process in an attempt to capture all the local differences between the clinical CT and atlas images. The generated b-spline transform matrix was applied to the atlas and its corresponding A-value fiducials, and the new distance between the fiducials was computed as the A-value of the target image.

### Evaluation of automated method

The registration algorithm was implemented on 19 specimens, as the atlas was excluded from the analysis. Depending on whether the target image was a right or left cochlea, the registration algorithm was applied using the corresponding atlas. The results of the automated method applied on the clinical CT images were compared to the gold standard A-values from the *μ*CT images of the same samples. Additionally, the automated method results were compared to the A-values manually acquired by experts on the same set of clinical CT images in a previous study [33].

### Qualitative evaluation

3D models of the atlas and a clinical CT image sample were created. The overlap of the models and the A-value fiducials were qualitatively evaluated before and after the registration algorithm. The deformation grids of the atlas, before and after each registration step, were also generated and visualized.

### Quantitative evaluation

A-values obtained using the automated registration-based method were compared to the gold standard reference values by calculating the absolute percentage difference. The mean percentage difference of the automated method from the gold standard was compared to the difference of the manual method reported in Iyaniwura et al. [33]. The automated and manually measured A-values were tested for normality using the Shapiro-Wilk test. Based on this result, the Wilcoxon matched pairs test was used to compare these values against the gold standard. The correlation between the two sets (automated and manual) of A-values and the gold standard A-values were evaluated using the Spearman correlation.

Lastly, Bland-Altman plots were used to display the differences between the A-values from clinical CT (automated method and manually measured) and the gold standard A-value measurements. A clinically acceptable A-value error range of ±1.05 mm was determined based on the revised cochlear length equations published by Koch et al. [18] and is indicated on the derived Bland-Altman plots.

## Results

### Qualitative results

The cochlear models generated from the *μ*CT (atlas), clinical CT (target), and the corresponding deformation grids were analysed. In all cases, affine registration successfully aligned the atlas and clinical CT images, addressing the majority of the global differences between the two images. Subsequently, the b-spline registration further improved the alignment addressing the local difference between the two images.

**Fig. 3:**
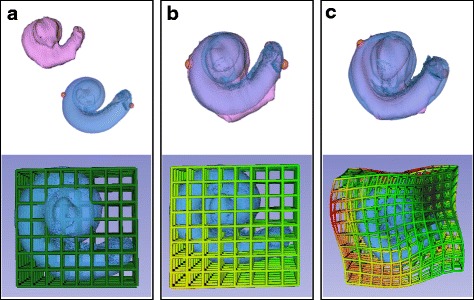
Micro-CT Atlas (blue) and Clinical CT Target (pink) before registration. **a** Original deformation grid around atlas shown. **b** Atlas and Target overlapped after affine registration with associated deformation grid. **c** Atlas and Target overlapped after B-Spline registration with associated deformation grid

Figure 3 provides a specific example where the target image was larger than the atlas. Affine registration globally expanded the atlas as shown by the deformation grid, and this achieved a partial overlap with the target (Fig. 3b). B-spline registration was then able to deal with the local differences, and the individual protrusions can be visualized on the deformation grid (Fig. 3c).

### Quantitative results

The absolute percentage difference (mean ± standard deviation), Wilcoxon test, and Spearman correlation were used to analyze manual and automated A-value measurements. Table 2 summarizes these results as compared to the gold standard measurements from *μ*CT. The automated method had a 2.7 ± 2.1% absolute difference from the gold standard compared to a difference of 9.5 ± 4.3% for the manual method reported in Iyaniwura et al. [33]. Using the Wilcoxon test, the automated method was not significantly different from the gold standard (*p* = 0.061, ns), but the manual method was significantly different from the gold standard (*p* < 0.0001). Comparing the automated method against the manual method, the results were significantly different from each other (*p* < 0.0001). Both the automated and manual methods had significant Spearman correlations of *r* = 0.70 (*p* < 0.01) and *r* = 0.69 (*p* < 0.01), respectively, when compared to the gold standard measurements.

**Table 2:** Percentage difference, Spearman correlation & Wilcoxon test comparison of manual and automated method

	% Difference	Wilcoxon	Spearman
Manual	9.5 ± 4.3%	*p* < 0.0001	*r* = 0.69**
Automated	2.7 ± 2.1%	*p* = 0.061 (ns)	*r* = 0.70**

**p < 0.01

**Fig. 4:**
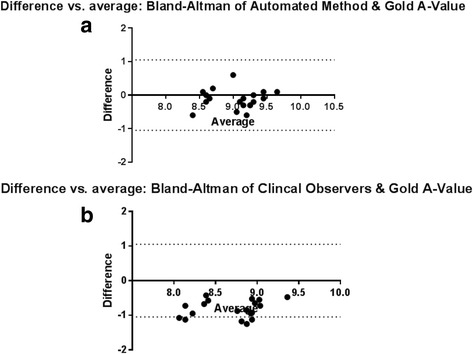
Bland-Altman plots of A-values estimated using the automated method (**a**) and manual measurements of A-value (**b**) in comparison to gold standard values. *Dotted lines at ± 1.05 mm represent the clinically acceptable error*

Bland-Altman plots were generated as shown in Fig. 4. A comparison of the automated method against the gold standard revealed that all measurements fell within the acceptable range (Fig. 4a). Experts’ manual measurements reported by Iyaniwura et al. [33] depicted an underestimation of true A-values. A second Bland-Altman plot was generated from these previously reported manual measurements and 26% of those measurements were found to be outside of the acceptable range (Fig. 4b).

## Discussion

As discussed, there is significant variation in cochlear size and morphology described in the literature [4–8, 42]. To develop a robust algorithm, 50 cochleae were initially scanned and a subset of 20 cochleae were chosen to represent a wide range of A-values. An additional strength of the study was the availability of corresponding *μ*CT images of the clinical CT images, which allowed for a gold standard validation of the algorithm.

Overall, the quantitative results revealed a statistically significant 6.8 ± 4.8% improvement in accuracy using the automated method. This algorithm also corrected the 26% of values that fell outside the clinically acceptable range using the manual method as observed on the Bland-Altman plots. The type of error on these plots was also different between the automated and manual methods. The automated algorithm had a random error centred on the origin, whereas the manual measurements consistently underestimated the true A-value (Fig. 4). The error observed in Fig. 4b can be described as a systematic error in the manual measurements. This error is most likely attributed to both the poor visibility of the round window in clinical CT images, and the variability associated with the selection of an oblique plane for the multiplanar reconstruction of each clinical CT image; however, a similar study with a larger n (participants would need to be conducted [33]. Furthermore the clinical CT images used in this study were of relatively low resolution (625um); however, other commonly used clinical CT scanning modalities such as cone beam computed tomography (CBCT), typically exhibit higher resolutions [43, 44]. As such an improvement in the algorithm’s performance would be hypothesized if utilized on CBCT images, for example.

Intra- and inter-observer variability between specialists also has been identified as a major source of error when measuring A-values on clinical CT [33, 34]. Iyaniwura et al. [19] reported intraclass correlation (ICC) coefficients for inter-observer variability (ICC = 0.57) and intra-observer variability (ICC range = 0.54 to 0.90). Rivas et al. [20] reported a mean absolute difference as high as 8 mm for CDL estimates calculated from manual A-value measurements. The automated algorithm described eliminates this observer variability.

The clinical significance of improved accuracy and consistency can be assessed by examining its effect on electrode selection and on the frequency mapping of the cochlea via the Greenwood equation. [16, 32, 45]. With regards to electrode selection, Iyaniwura et al. [33], using an average CDL value of 32.9 mm, derived an average CDL variation of ±3.9 mm with the manual method. Cochlear implant manufacturers have off the shelf implants available in 15, 17, 20, 24, 25, 28 and 31 mm variants, therefore a variation of ±3.9 mm could lead to improper electrode selection preoperatively [13, 46–49]. In terms of customized frequency maps, Koch et al. [14] calculated that a 6 mm error in CDL would result in a frequency-place mismatch of 400 Hz at the apical turn and 1100 Hz along the basal turn of the cochlea. These discrepancies could translate into discernible effects on cochlear implant performance [15, 24].

There have been a number of registration techniques that have been described in the medical imaging literature. In structures with significant variability, FFD (non-linear/non-rigid) registration like b-spline, as well as atlas-based registration techniques, are typically used [39, 41, 50–56]. In this study, a single atlas with b-spline registration was sufficient in improving the accuracy of A-value measurements, which are based upon the basal turn of the cochlea. However, the cochlear apex can exhibit significant additional variation between patients [5, 14, 32, 45] If the apex was to be directly modeled in the future, this could be overcome using multi-atlas registration techniques. [56–58].

Other studies have attempted to register inner ear structures for a variety of purposes. Christensen et al. used a deformable atlas based registration technique to measure shapes within the inner ear [59], however they did not measure the A-value or the CDL. Rivas et al. [34] developed a sophisticated algorithm for measuring the CDL, however no high-resolution *μ*CT images were available to validate their results. The A-value measured by the algorithm can then be used to estimate the CDL at the lateral wall and Organ of Corti by using equations developed by Alexiades et al. [32] and Koch et al. [14]. These equations have been built into the module as part of the output to the end-user.

The implemented automated algorithm will be made available as an open-source software extension to 3D Slicer. This would allow for further development by other groups and validation of the methodology on a wider variety of cochleae.

## Conclusion

An automated method to estimate cochlear length based on the A-value was developed using open-source atlas-based registration tools. The automated method produced more accurate results than the manual method, and eliminated the observer variability between experts. This improved accuracy may be clinically important for electrode selection and patient-specific frequency mapping of cochlear implants.

## Abbreviations

3D: Three dimensional
CBCT: Cone beam computed tomography
CDL: Cochlear duct length
CI: Cochlear implants
CT: Computed tomography
DOF: Degrees of freedom
FFD: Free-form deformation
ICC: Intraclass correlation
NCC: Normalized cross correlation
SNHL: Sensorineural hearing loss
*μ*CT: Micro-CT
